# A Clinical Perspective of Low Carbohydrate Ketogenic Diets: A Narrative Review

**DOI:** 10.3389/fnut.2021.642628

**Published:** 2021-07-12

**Authors:** Samir Giuseppe Sukkar, Maurizio Muscaritoli

**Affiliations:** ^1^Unità Operativa Dipartimentale Dietetica e Nutrizione Clinica, Dipartimento Medicina Interna, Policlinico San Martino di Genova Istituto di Ricovero e Cura a Carattere Scientifico per l'Oncologia e la Neurologia, Genova, Italy; ^2^Unità Operativa Complessa di Medicina Interna e Nutrizione Clinica, Dipartimento ad Attività Integrata di Medicina Interna Scienze Endocrino-Metaboliche e Malattie Infettive, Azienda Ospedaliera Universitaria Policlinico Umberto I, Rome, Italy

**Keywords:** low-carbohydrate diet, ketogenic diet, obesity, fasting, protein sparing modified fasting, fast-mimicking diet, low calorie diets, very low calorie diet

## Abstract

Low carbohydrates diets (LCDs), which provide 20–120 g of carbohydrates per day, have long been used as therapeutic options in the treatment of severe obesity, type 2 diabetes mellitus and other morbid conditions, with good results in terms of weight loss and control of the main metabolic parameters, at least in the short and medium term. According to the caloric content and the macronutrient composition, we can classify LCDs in hypocaloric, normoproteic diets [such as the Very Low-Calorie Ketogenic Diet (VLCKD) or the protein-sparing modified fasting (PSMF)], hypocaloric, hyperproteic and hyperlipidic diets (e.g., Atkins, Paleo diets…) and normocaloric, normo-/hyperproteic diets (eucaloric KD), the latter mainly used in patients with brain tumors (gliomas) and refractory epilepsy. In addition to LCD diets, another interesting dietary approach which gained attention in the last few decades is fasting and its beneficial effects in terms of modulation of metabolic pathways, cellular processes and hormonal secretions. Due to the impossibility of using fasting regimens for long periods of time, several alternative strategies have been proposed that can mimic the effects, including calorie restriction, intermittent or alternating fasting, and the so-called fasting mimicking diets (FMDs). Recent preclinical studies have shown positive effects of FMDs in various experimental models of tumors, diabetes, Alzheimer Disease, and other morbid conditions, but to date, the scientific evidence in humans is limited to some opens studies and case reports. The purpose of our narrative review is to offer an overview of the characteristics of the main dietary regimens applied in the treatment of different clinical conditions as well as of the scientific evidence that justifies their use, focusing on low and zero-carb diets and on the different types of fasting.

## Low Carbohydrate Diets

The use of low or zero carbohydrate diets has long been a therapeutic option in various morbid conditions. Although over the years there has been a fluctuating position, sometimes unfavorable and sometimes favorable, regarding their use in clinical practice, currently, in light of the evidence of the literature, it finds more and more evidence in its favor, but only in certain clinical conditions (1–18).

It is useful to clarify that the term “high-protein diets,” often used in reference to low-carbohydrate diets, is incorrect because diets characterized by the reduction of the carbohydrate load can also be normo-protein diets.

From a metabolic point of view, low calorie (LCD) diets with low carbohydrate content (20–120 g of carbohydrates/day), which provide 1,000–1,200 calories per day, are indicated in the treatment of obesity as they promote a reduced increase in insulin and an increase in glucagon, which generates greater oxidation of fats (1). However, despite the theory of the insulin carbohydrate model, clinical trials that compared LCD with low fat-isoproteic diets (LFD) reported similar weight loss (2, 3) and fat loss higher when lipid intake but not carbohydrates are reduced (4). Furthermore, a meta-analysis of 32 controlled studies shows that energy expenditure and fat loss are considerably higher with LFDs than isocaloric LCDs (5).

Low calorie diets below 30–50 g of carbohydrate content that causes ketosis and mimics the physiologic state of fasting are called ketogenics: Low Calorie ketogenic diet (LCKD).

With regard to macronutrients, the difference between these diets depends on the percentage of residual calories from fats (hypo, normal or hyper lipids) and proteins.

Low calorie ketogenic diet (LCKD) are diets with carbohydrate intake <30 g/day (13% of the total energy intake), with a relative increase in fats (44%) and proteins (43%) and a total daily energy intake of 800–1,200 (3, 7, 13).

Very low calorie ketogenic diet has an energy intake of <800 cal, with a daily protein intake of about 1.2–1.5 g/kg of ideal body weight (8–14).

Protein sparing modified fast (PSMF) differs because the main source of calories are protein and not fat and therefore the calorie intake is generally lower and corresponds to 400 calories per day, always with a protein intake of 1.2–1.5 g/kg/day of protein (15–21). Carbohydrates are limited to <20–30 g per day. Therefore, VLCKD and PSMF can be considered semi-fasts slightly hyperproteic.

From the point of view of the composition in macronutrients, the difference between these diets is dependent on the percentage of residual calories from fats (low, normal or high lipids) and proteins (low, normal or high proteins).

From a systematic perspective, we can differentiate:

1) Normoproteic low calorie diets (LCD) and low calorie Ketogenic diets (LCKD);
2) normoproteic very low-calorie carbohydrate, hyperlipidic diets [very low calorie Ketogenic diets (VLCKD)];
3) normoproteic, very low-calorie carbohydrate, hypolipidic diets (protein-sparing modified fast);
4) normo or hyperprotein, normocaloric, low carbohydrate diets (eucaloric ketogenic diets) (EKD);
5) hyper-protein, low-calorie, low-carbohydrate, hyperlipidic diets (e.g., Atkins, Scarsdale, Planck, Palaeo)

Each of these types of diets has different pathophysiological bases that provide specific therapeutic indications except for hyperlipidic, protein-rich, low-calorie diets. These diets are related to greater short-term weight loss. Anyway, they are not currently suggested as they could present negative effects on metabolism and intestinal microbiota and certainly cannot be proposed as a healthy lifestyle food model (7, 22–24).

**Figure 1:**
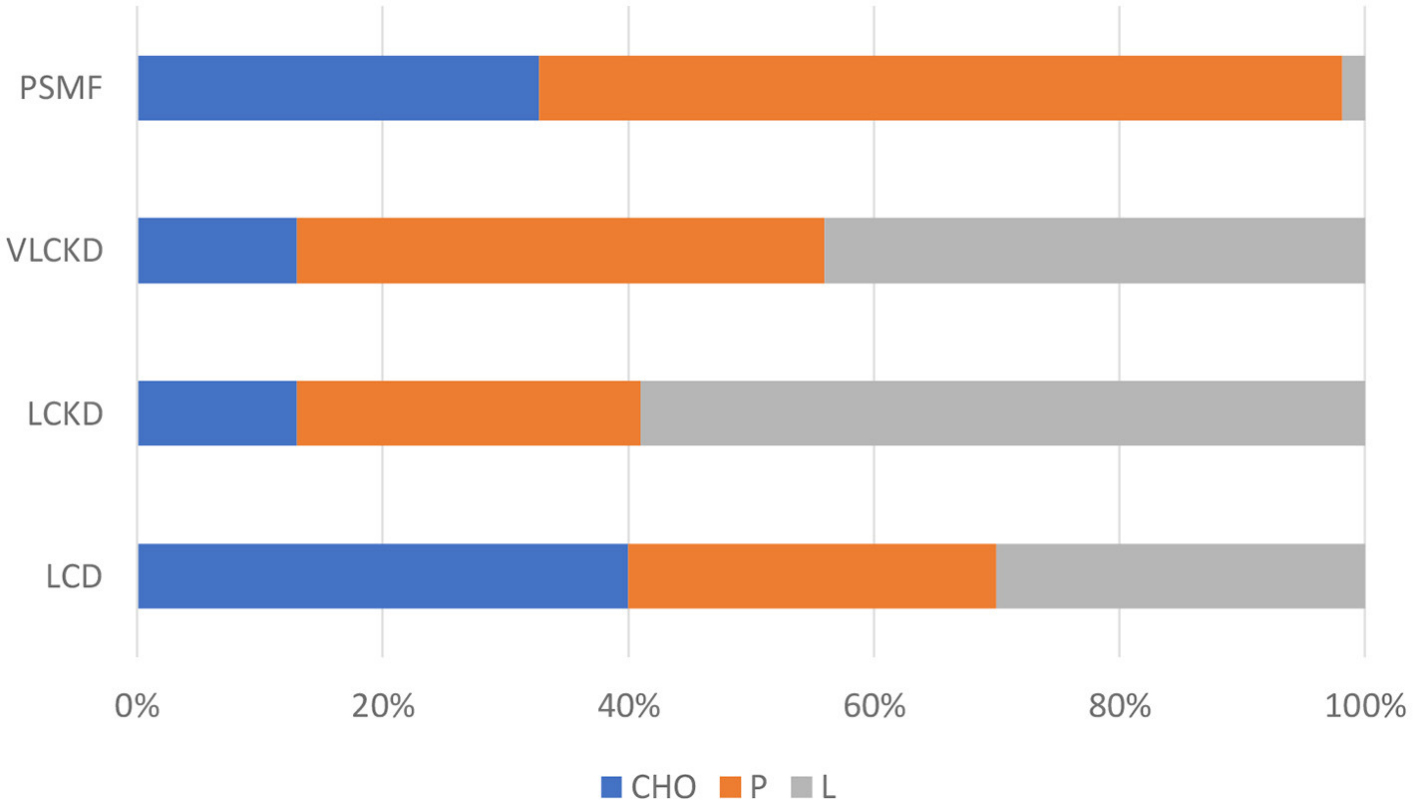
Approximate macronutrient percentage in low calorie diets.

The characteristics and indications proposed for this type of diet are summarized in Table 1, while the approximate percentage of macronutrients is indicated in Figure 1.

**Table 1:** Classification of low carbohydrates diets and strength of the recommendations for their use.

**Classification of low carbohydrate diets**	Strength of recommendations and quality of evidence according to the GRADE system^*^			
	**1 ØØØO**	**2 ØØØO**	**2 ØØOO**	**2 ØOOO**
Normoproteic hypoglucidic diets: Low calorie diets (LCD) and low calorie Ketogenic diets (LCKD)	• Obesity BMI 25–35 (hypertension, type 2 diabetes, dyslipidemia, OSAS, metabolic syndrome, osteopathies or severe arthropathies) • Obesity associated with type 2 diabetes • Obesity associated with hypertriglyceridemia • Obesity associated with hypertension • Pediatric obesity associated with epilepsy and / or a high level of insulin resistance and / or comorbidity, not sensitive to the standardized diet	• Obesity associated with intestinal microbiota dysbiosis • Obesity associated with high levels of LDL cholesterol and / or low levels of HDL cholesterol • Obesity associated with non-alcoholic hepatosteatosis (NAFLD) • Male obesity associated with hypogonadism		• Obesity associated with heart failure (NYHA I - II) • Obesity associated with atherosclerosis • Obesity associated with polycystic ovary syndrome (PCOS) • Obesity linked to the transition of menopause • Neurodegenerative disorders associated with sarcopenic obesity
Normoproteic very low calorie hypoglucidic diets • Very Low calorie diets (VLCKD) • protein-sparing modified fast: PSMF);	• Severe or complicated obesity (hypertension, type 2 diabetes, dyslipidemia, OSAS, metabolic syndrome, osteopathies or severe arthropathies) • Severe obesity with indication for bariatric surgery (in the pre-operative period) • Patients with rapid weight loss indications for severe comorbidities • Obesity associated with hypertriglyceridemia • Adolescents with severe obesity			
Normo- or hyper-proteic hypoglucidic diets (eucaloric ketogenic diets, EKD)	• epilepsy resistant to antiepileptic therapy		Glioma and glioblastomas	• Neurodegenerative diseases (Alzheimer's disease, Parkinson's disease), • Neurocognitive disorders (Mild cognitive impairment, MCI), • Brain trauma (Traumatic brain injury, TBI)

* *GRADE system = Classification of quality of evidence and strength of recommendation*.

### Low Calorie Ketogenic Diets

Ketogenic diet in practice is a very low carb diet with a variable fat content and usually normoproteic. In addition, different therapeutic modalities and specific variants are distinguished according to the clinical purpose: obesity, neurological pathologies, congenital metabolic pathologies, etc.

While treating obesity, ketogenic diets differ according to the calories introduced:

Low Calorie Ketogenic Diets (LCKD), Very Low Calorie Ketogenic Diets (VLCKD), modified protein-saving fast (PSMF).

#### Low Carbohydrates Hypoproteic Low-Calorie Diets (Low Calorie Ketogenic Diets and Very Low Ketogenic Diets)

Over the past decade, a lot of studies have documented evidence of the therapeutic efficacy of LCKD, in obesity (2–4), associated or not with comorbidity and in the preparatory phase for bariatric surgery (7–9) (Table 1).

A VLCKD, according to the guidelines of the European Food Safety Authority 2015 *, provides for the intake of <800 calories per day, a protein content of 1.2–1.5 g/kg of ideal body weight, a minimum amount of carbohydrates <30 g/day, a fat percentage of ≃44%, a minimum content of linoleic acid equal to 11 g/day and α-linolenic acid equal to 1.4 g/day, and vitamins and minerals equal to the daily needs (10–12). In addition to the low calorie intake, the main characteristic of a VLCKD diet is that it provides a reduced carbohydrate intake that stimulates the lipolysis of the storage fat and determines a physiological ketosis. The ketosis that occurs during a VLCKD always remains moderate (ketonemia never exceeding 3 mMol/L) and constitutes a physiological mechanism of energy control widely used by man in any situation of reduced glucose intake.

This ketosis is completely unlike diabetic ketoacidosis, characterized by: hyperglycemia [blood glucose (BG) > 250 mg/dl]; anion gap metabolic acidosis (pH < 7.30 and bicarbonate < 18 mEq/L); and high ketonemia which reaches 15–20 mMol/L, therefore 5–10 times higher to those of nutritional ketosis.

According to international guidelines, a VLCKD can be used continuously for up to 12 weeks, but it must always be performed under medical surveillance (7–9).

This type of diet achieves the desired weight in less time than conventional low-calorie diets. Usually, an average weight loss of 1–1.5 kg per week is achieved with variations due to gender, body type and individual physical activity.

In addition, with this diet there is an interaction between the satiating effect of proteins and the presence of ketone bodies derived from the use of storage fats, in a better appetite control, always present in traditional low-calorie diets, which is greatly attenuated starting from 36 to 48 h.

In the VLCKD, the use of industrial meal replacements is often used, which may allow greater safety with respect to food components, well-quantified and better balanced (8, 9, 11).

Of course, VLCKD is a transitional method after which, gradually, the return to a correct food style, traditionally based on an accurate balance between the various nutrients: carbohydrates, proteins and fats, must be followed.

Recent studies have demonstrated the validity of VLCKD in comparison with low-carbohydrate (LDC) non-ketogenic diets. In particular, Moreno et al. (13) conclude that VLCKD is well-tolerated and moderate and has transient side effects, and is more effective than a standard very low calorie diet (VLCD). After a year of follow-up, lean body mass was well-preserved among subjects who had lost more than 10% of their initial weight (13); equally Merra et al. (14) showed that a VLCKD was highly effective in terms of reducing body weight without inducing loss of lean mass, thus preventing the risk of sarcopenia (14). Therefore, muscle mass is not affected, but it could be maintained by adequate protein supply.

#### Normoproteic Low Carb Diets (CHO < 30 g/day): Protein Sparing Modified Fasting

The PSMF diet was developed in 1970 by the working group led by Bistrian and consists in the administration of only proteins for a contribution of 1.2–1.5 g/kg (ideal body weight)/day with supplementation of vitamins and minerals (15). This diet, if controlled in a medical environment, allows excellent results to be obtained even with long-term weight reduction maintenance (16–18) (Table 1). It has recently been shown that the PSMF diet can be used as an effective and safe outpatient method for rapid weight loss in adolescents with severe obesity (19). The calories introduced with this type of diet are very limited, usually < 400 kcal/day. From a nutritional point of view, this diet is not considered complete and, for this reason, nutritional supplementation is necessary. The PSMF regimen in fact involves the intake of vitamins and minerals, such as a multivitamin and 2–3 g of potassium, to compensate the lack of micronutrients due to the scarcity and limited supply of food (18). Furthermore, the consumption of at least 2 liters of calorie free liquid per day is expected (20). From the caloric point of view, the main source is a minimum amount of fat (20 g) in order to reduce the risk of cholelithiasis while the carbohydrate quota is <20 g generally ensured by the use of vegetables, while saving protein is represented by proteins that are supplied in the amount of 1.2–1.5 g/kg on the ideal weight which are actually used for energy in the first 36–48 h of metabolic shift toward ketosis and subsequently for plastic purposes (15). The proteins used are high quality proteins, to a degree that prevents or significantly reduces skeletal muscle loss. Body fat losses correspond to about 0.2 kg/day for women and 0.3 kg/day for men, and therefore in 6 weeks it is possible to obtain an average reduction of 14 kg of fat, limiting the loss of lean mass (21). The benefits of PSMF are not limited to the loss of body fat, but can also include an improvement in blood pressure, blood sugar and lipids (18). With regard to the possible side effects of the PSMF, various aspects should be considered. Since PSMF is a normoproteic diet, no risk of kidney damage is expected in both young and elderly subjects who are unable to respond to the protein increase above 2.5 g/kg/day with an increase in glomerular filtrate (22, 23). For the same reason, there are no risks of stone formation resulting from the acid-base imbalance in calcium metabolism as this risk is observed only with diets with high protein quotas (>2 g/kg/day) also associated with high energy supplies (24). In fact, PSMF could, on the other hand, be involved in improving kidney function and Poplawski et al. have actually shown, in mouse models, that a ketogenic diet regresses, even in histological terms, the process of diabetic nephropathy (25). The authors believe it is plausible that the use of a ketogenic diet for a limited period of time would produce a sustainable regression of the underlying conditions associated with diabetes, significantly resetting gene expression profile (25).

With regard to other possible side effects of the PSMF, various aspects should be considered. In terms of liver complications, it has been known since 1992 that both mild portal inflammation and fibrosis and the risk of gallbladder formation may occur following PSMF. However, subsequent data indicate that to avoid biliary stasis, due to the reduced motility of the gallbladder during PSMF, it is sufficient to introduce a minimum fat content of 10 g/day (26). Concerning the risk of osteoporosis, associated with the increase in calciuria due to acidosis linked to a high protein intake (which does not really exist in this type of diet). According to the Bonjour review, there is no causal relationship between animal proteins and increased incidence of osteoporosis fractures (27). In addition, the increased calciuria that can be observed as a result of increased protein intake from animal and plant sources can be explained by the stimulation of intestinal calcium absorption. It should also be noted that dietary proteins increase IGF-1 which exerts a positive action on bone development and formation (27). However, there is not enough evidence to argue that the benefit of protein on bone leads, in the long run, to a reduction in the risk of osteoporosis fractures (28). Finally, there is one important caveat in the literature: observations of certain deaths that have been observed as a result of ventricular arrhythmias in patients with extended periods of PSMF (16). These deaths have been proven to be the result of the use of hydrolysed collagen proteins and in addition to the lack of integration with ions and vitamins (16). It is therefore essential that a modified fasting protocol with a protein content of at least 1–1.5 g/kg/day, with 20 g of lipids, can be with adequate vitamin, hydroelectrolytic and fiber (20 g/day) and followed under close specialist medical supervision.

### Normocaloric Ketogenic Diets (Hyperproteic and Normoproteic) in Pathological Conditions (Not for “weight Loss” Purposes)

Hyperproteic and normoproteic ketogenic diets (KD) have long been used in the treatment of pharmacoresistant epilepsy. In more recent times, some preclinical and clinical evidence support the use of ketogenic diets also in other areas, such as neurodegenerative diseases (Alzheimer's disease, Parkinson's disease), neurocognitive disorders (Mild cognitive impairment, MCI), brain trauma (Traumatic brain injury, TBI) even if the most interesting data come from their use in the neuro-oncological field.

#### Preclinical Evidence: Experimental Models of Brain Tumors and Ketogenic Diet

**Figure 2:**
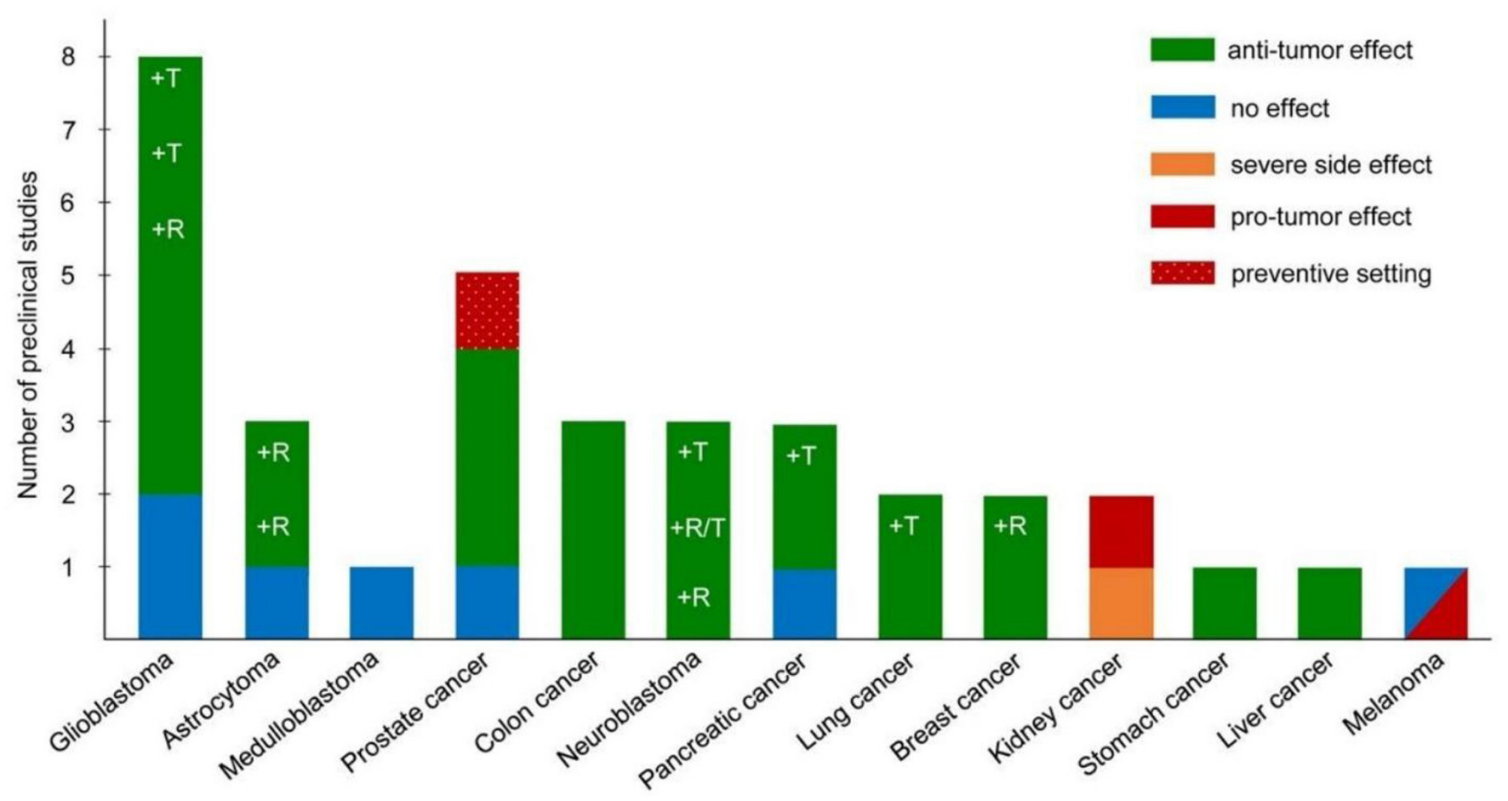
Pre-clinical studies on the ketogenic diet as antineoplastic therapy, adapted (29).

There are several studies published on experimental models of different types of malignant neoplasms, treated with the ketogenic diet or in combination with standard therapy (radio and chemotherapy) (Figure 2). Without entering into the details of each individual study and the type of neoplasm taken into account, the effects are globally positive on the survival or reduction of the tumor mass, with some exceptions concerning some melanoma and renal cell lines where the ketogenic diet has a promoter effect against the cancer (30, 31). Several therapeutic mechanisms exist: the reduction of the angiogenic process of the neoplasm, the radiosensitization, the chemosensitization in particular to the PI3K inhibitors, the reduction of the inflammatory processes of the tumor microenvironment and the conservation of the lean mass.

The promoter effects are unclear, as different tumors with similar alterations in mitochondrial oxidative metabolism have produced opposite results with such diets, as in the case of gliomas (anti-neoplastic effect) and renal carcinoma (pro-neoplastic effect). In a breast cancer model, although the neoplastic cells had an enzyme kit capable of using ketones (BDH-1 and OXCT-1, which convert ketone bodies back to Ac-CoA), there was no promoter effect of ketones. On the other hand, an antineoplastic effect has not even been observed (32).

Considering studies on glioma and astrocytoma models, the most used metabolic treatment is the classic 4: 1 or 3: 1 ketogenic diet, *ad libitum* or energy-restricted. Studies were conducted with both the ketogenic regimen as a unique therapy and in combination with chemotherapy (CT) or radiation therapy (RT). Although the premises based on *in vitro* models have been very optimistic about the antineoplastic role of beta-hydroxy-methyl-butyrate (BHB) or of aceto-acetate, the results are not always as expected *in vivo*. Taking into account the experimental models with control group (KD vs. standard diet), the antineoplastic effect of KD does not always occur, while an improvement in survival is evident, and in one study, the complete remission of the tumor when it was associated to RT or CT. The effect also occurs in the absence of traditional RT or CT (Temozolomide) if it is administered with metabolism antagonists of glutamine (6 - diazo - 5 - oxo - L - norleucine, DON) or glucose (2-deoxyglucose), indicating how these models of gliomas are greatly influenced by the host's metabolic and nutritional status (33, 34).

#### Clinical Evidence of the Ketogenic Diet in CNS Neoplasm

The available human studies are currently very heterogeneous because there is no standardized therapeutic protocol. Ongoing clinical trials seem to favor modified ketogenic diets (MKD), with a less extreme ketogenic ratio (3: 1, 2: 1 or variable as in the modified Atkins diet) and integration of nutritional supplements with MCT to facilitate ketogenesis and l adherence to the diet protocol. Besides anecdotal studies, most prospective studies are open.

The most recent prospective study considered patients with glioblastoma multiforme (GBM) (WHO grade IV) who underwent surgery and post-operative CT and RT. Nine patients were selected, of whom 6 concluded the study period (14 months, of which 12 in ketosis) (35). Before starting the RT and CT, they were started to a classic 4: 1 normal caloric KD protocol with liquid formula rich in MCT, upon reaching a plasma BHB level of 3 mM they switched to a solid diet with MCT integration (70%) to maintain high ketonemia. Quality of life and neurological aspects did not change during the study and the average survival was 12.8 months (8–19 months), in line with what was expected in the absence of a ketogenic diet (11–13 months), however as reported the authors, the sample is very limited and with great variability of survival. The authors stressed, as already described by Schwartz et al. (36), the need for continuous dietary follow-up, to solve any nutritional problems, adherence and above all to help the patient and family members to remain motivated to follow the diet protocol (35). The only clinical trial published to date, is a pilot study conducted on 20 patients with recurrent GBM, including 8 on the modified ketogenic diet with MCT drink without calorie restriction. Patients were monitored and the ketogenic regimen showed higher latency to tumor growth recovery (progression-free survival) compared to untreated patients (median 6 vs. 3 months). The patients, following the progression of the neoplasm, resumed a cycle of CT (Bevacizumab, anti VEGF drug) maintaining the ketogenic diet and arriving at a survival median of 20.1 months, compared to 16.1 months of a similar cohort of patients treated with the only drug (37). According to a systematic review of the literature on clinical and pre-clinical studies, KD is effective and safe in experimental models, and of possible benefit and safe in patients as adjuvant and neo-adjuvant therapy for the control of neoplasm and associated complications of type neurological. A number of clinical trials are underway to verify safety, compliance and efficacy in enhancing survival (34). Table 2 summarizes the clinical trials.

**Table 2:** Published clinical studies on Eucaloric Ketogenic diet in Neurological cancers, adapted from (30).

**Type of cancer**	**Study group**	**Dietary intervention**	**Study completion (*n*)**	**Cancer therapy**	**Study duration**	**Primary outcomes**
Glioblastoma multiforme (GMB)	1	CR-KD 4:1 600kcal	01-gen	ST	14 days KD-CR, 5 months CR	Complete response at 2 months, recurrence at 10 weeks post CR suspension
Glioma	20	KD with drink MCT, 60g CHO	ago-20	ST	6 weeks	Trend for un greater PFS (*n* = 8) with 1 complete response, 5 PR
Glioma	2	CR-KD 3:1 20% CR	01-feb	No	3 months	TP in both patients
Primary malignant brain tumors	8	MAD 20g CHO	07-ago	ST (3/8)	2–24 months	Better control of epileptic crisis in all patients alive at the 13th month of follow up
Glioblastoma	32	KD 50% kcal fat, 25%kcal CHO, 1.5 g/kg protein (17), CD (15)	set-17	55 mg Perillic intranasal alcohol	3 months	KD group: 78% PR, 11% SD, 11% TP. CD group : 25% PR, 25% SD, 50% TP
Glioblastoma	1	CR-KD 4:1 900 kcal pre-operative. Then 1,500 kcal	01-gen	CT + RT + other drugs + oxygen therapy	9 months	Regression of the tumor. After 20 months regression continues
Glioblastoma	53	KD 30–50 g CHO	06–giu	RT (4/6)	3–12 months	5 TP, 1 complete response 12 months post-RT
Glioma	172	MKD 70% FAT, 20 g CHO	04-giu	ST	3 months	No data on the neoplasm. Well tolerated diet
Malignant Astrocytoma tumors	2 (children)	KD 70% FAT	02-feb	ST	8 weeks	Complete answer. Remission of the disease for 5 and 4 years

*KD, ketogenic diet; CR, calorie restriction; ST, standard therapy; PFS, progression-free survival; PR, partial response; TP, progression; SD, stable disease*.

The protocols used are still very heterogeneous with one another, both in terms of ketogenic ratio and calorie intake. The choice of the protocol in addition to ensuring adequate ketosis, requires high patient adherence. This aspect is actually essential for benefiting from the metabolic modulation aspect on healthy tissues and for enhancing the effects of therapy on the tumor.

### Low-Calorie, Low Carb, Hyperlipidic, Hyperproteic Diets

Scientifically verified low-carbohydrate diets include the Atkins diet (such as Plack, Scarsdale, etc.) and the Paleolithic diet. The most used variant in clinical practice is the one called Atkins diet. This regimen provides an initial “ketogenic” period where carbohydrates (<10–15g/day) (VLCKD) are strongly restricted in favor of a fairly liberalized consumption of various protein foods. Therefore, there is a spontaneously reduced caloric intake due to anorectic effect of ketones and proteins. Endogenous lipolysis provides the necessary Ac-CoA for ketogenesis (38) associated with a high amount of lipids. Popular low-protein high-protein diets, such as Atkins or Zone, produce a significant weight loss in the short term (39, 40), increasing satiety and energy expenditure and body composition (41). On the other hand, in the long term (in clinical trials longer than 1–2 years) there are no differences in weight loss (39, 40, 42–44). Furthermore, hyperlipidic high-protein diets have a higher intake of saturated fats and animal proteins and are associated with an increase in LDL cholesterol values LDL cholesterol (5, 45–48). The Paleolithic diet, also called Paleo or Paleodiet, is based on foods of daily use that imitate the food groups of the Paleolithic hunter-gatherers (49, 50). The diet is high in protein (20–35% energy) and moderate in fats and carbohydrates (22–40% energy, high glycemic index), low omega-6/omega-3 ratio low in sodium, together with a high content of unsaturated fatty acids, antioxidants, fibers, vitamins, and phytochemicals (51). Paleodiet trials showed positive effects in metabolic syndrome (52), increased insulin sensitivity (53) reduced cardiovascular risk factors (54, 55), increased satiety (56, 57) and beneficial modulation of the intestinal microbiota (58). In particular, regarding the Paleodiet for weight loss, scientific evidence indicates a constant reduction of body weight and body fat mass in short (55, 59–61) or long-term studies (62, 63). Moreover, poor adherence over time (59), poor palatability, the presence of a potential risk of deficiency which includes vitamin D, calcium (60) and iodine (64), and high costs are important factors limiting the use of this diet (65).

In conclusion, in the short term, hyperlipidic diets with high protein content with low carbohydrate content show greater effectiveness in terms of weight loss. Furthermore, negative effects on the metabolic level and the microbiota could be considered as a short-term therapeutic tool, but not as a dietary model for life. In addition, in the long term, it has been shown that diets with a different macro-nutrient composition do not have a different efficiency in weight loss.

## Fasting Regimes

Apart from the physiological fasting, many treatments have tried over time to achieve the benefits that the fasting condition produces. The basic purpose of fasting is to promote changes in metabolic pathways, cellular processes and hormone secretions (66). The major physiological responses of fasting on health indicators include better insulin sensitivity (67), improved blood pressure levels (68), reduced body fat (69), blood sugar (70), atherogenic lipids (71) and inflammation (72). In the animal model, fasting is associated with interesting outcomes in correcting type 2 diabetes (73) and cardiovascular disease (74). In humans, 12–24 h of fasting are associated with a significant 20% reduction in blood glucose and hepatic glycogen depletion. Under these conditions, the transition to ketogen metabolism occurs (66). In oncology, in preclinical studies, 48-h water fasting is able to prevent DNA damage in healthy cells, usually induced by chemotherapy agents but, in addition to being difficult to accept and completed by patients, it can cause macro and micro-nutritional deficiencies (75–78).

### Alternatives to Fasting

The alternative to fasting is represented by continuous calorie restriction, an approach that involves reducing 20–40% of calories continuously, which can cause numerous side effects such as irritability, depression, obsession with food and in cancer patients can lead to malnutrition, a very dangerous condition in this type of patient (78–82). For this reason, another method of daily calorie restriction can be carried out by manipulating meal times and frequencies over time (daily, weekly, monthly). This method refers to calorie restriction and therefore the length of fasting between meals to establish a new kind of strategy.

#### Intermittent Fasting

Intermittent fasting is the most cited among these methodologies and consists in abstaining from food and calorie drinks for a certain period of time (83, 84). Different variants of intermittent fasting differ in the duration and frequency of fasting cycles. In addition, modified intermittent fasting allows small contributions from energy foods to reduce hunger stimulation (85).

**Figure 3:**
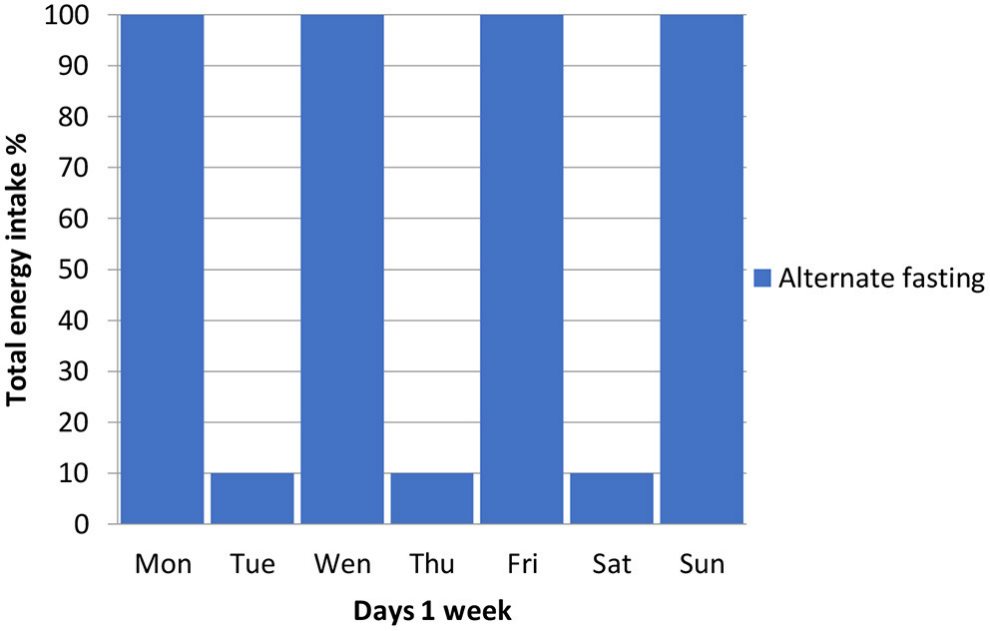
Alternating 1:1 fasting.

**Figure 4:**
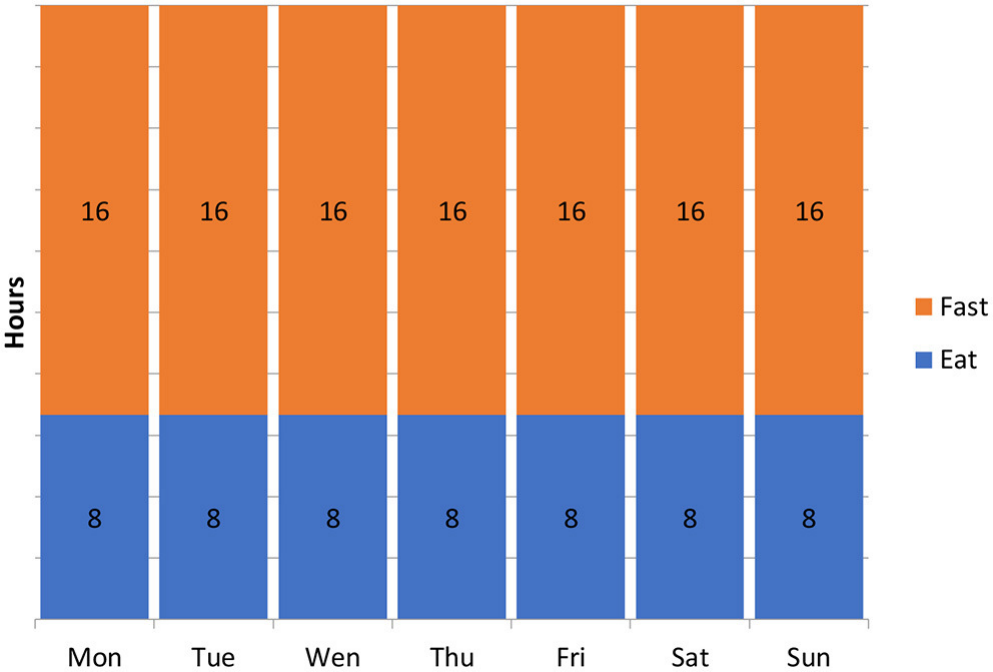
Hourly fasting 16:8.

The most common types of intermittent fasting include periodic 5: 2 fasting, alternating 1:1 fasting (Figure 3), hourly food restriction (e.g., 16: 8) (Figure 4), and religious fasting (67–70, 74, 85–99).

In the experimental animal the effects are contradictory: in mice, alternating fasting is not able to reduce muscle insulin resistance induced by high fat diets and is not able to promote changes in weight loss (70, 86, 89).

In humans, although in the short term intermittent fasting allows an average weight loss of 4–10% in periods of 4–24 weeks (68, 69, 72, 87, 88, 100, 101) in the few studies lasting >6 months the reported results they are modest (68, 88, 102–104). In several clinical studies, the absence of adequate control groups suggests that intermittent fasting has not yet been rigorously studied in the long term. Two recent meta-analyses provided a summary of the effects of intermittent energy restriction in intervention studies (84, 105). Both analyses found that no intermittent or continuous calorie restriction was greater than the other for weight loss. Moreover, in a recent comparative real life study of the Mediterranean diet, Paleo diet and intermittent fasting for a period of 1 year, it has been observed that the Mediterranean diet and intermittent fasting give similar results in terms of weight loss but the Mediterranean diet allows a greater benefit in the glycemic control in relation to the consumption of plant foods with a higher fiber content (83). Finally, as regards religious fasts, the data are inconclusive: as regards Ramadan, several studies have reported weight loss (106, 107), while others have not shown any significant changes (90, 108, 109). Very often weight recovery is observed a few weeks after the fasting period (91, 107, 110) while weight loss for daily calorie restriction periods is observed but the results are still short-term. Finally, it should be stressed that fasting without adequate protein content can be harmful for some populations such as children, the elderly and underweight patients.

#### Fast-Mimicking Diet

The Fast-mimicking diet (FMD) is a dietary regimen, to be carried out under strict medical supervision, which provides for a very low calorie intake (300–1,100 kcal/day), as well as low is the introduction of carbohydrates and proteins in order to mimic water fasting but with possible better compliance by patients (75–77). Patients receive standardized portions of vegetable broths, soups, juices, dried fruit bars, herbal teas and in addition micronutrient supplements. Protocols provide that fast-mimicking diet is observed for 2–5 consecutive days a month. Several studies have shown efficacy in the experimental animal in various morbid conditions such as diabetes and tumors, but in humans the data are limited to open studies or case reports. The only randomized phase 2 study is a cross over that compared a group treated with three cycles of FMD with a group treated with a normal calorie diet (111). Subsequently, the group with normocaloric diet was treated with 3 cycles of FMD and the FMD group with control diet, which also was normocaloric. The result is easy to interpret: with the cross over it simply doubled in number of patients but the comparison between FMD remained the normocaloric diet. Obviously it is paradoxical to think that a fast is compared with a normocaloric diet because the results are necessarily spurious.

The absence of comparison between a low-calorie diet and a FMD is a severe bias. As a matter of facts the study demonstrated that a 3 cycles of fasting reduce body weight, waist circumference, BMI, total body and trunk fat, systolic blood pressure and IGF-1 compared to a normocaloric diet. But not only, although there is no real control group, the authors also point out that, from a *post-hoc* analysis, the subjects who had high risk factors or metabolic markers associated with metabolic syndrome and age-related diseases (such as a high body mass index, elevated blood pressure, high blood glucose levels, triglycerides, CRP, cholesterol and IGF-1), were significantly improved compared with non-risk individuals (111).

In fact, the same results could be observed with any low-calorie regimen conducted for the same period, even more on high-risk individuals, where it is well-known that weight loss is related to the reduction of metabolic risk factors and pressure.

Therefore, the study does not yield any actual conclusions or indications.

Consequently, it is necessary that rigorous studies are conducted with real control arms with low calorie diets. This study simply strengthens the evidence for calorie restriction in preventing chronic degenerative diseases, malignant tumors and longevity.

As regards tumors, several preclinical studies have shown that fasting diets or fast-mimicking diets exert powerful anticancer effects in experimental animals, both in solid tumor models (such as breast, lung, and gliomas) and in hematological tumors. These dietary approaches can reduce nutrient levels/factors that promote proliferation, particularly glucose, IGF1 and insulin, increase ketones body level which help to slow tumor growth and promote antitumor immunity and sensitization of cancer cells to the action of the immune system (112, 113).

By contrast, fasting, unlike other dietetic approaches, induces “starvation” modality in cells.

In fact, fasting can activate a sustained evolutionary molecular response to metabolic stress in normal cells, inhibiting their proliferation, increasing the maintenance of self with effect of protection from chemotherapy and toxic agents (114). This mechanism can be noteworthy if we consider the damage often caused by these drugs, the side effects of which can be serious or even lethal for the lesions suffered by epithelial and non-epithelial tissues. In this way, it is possible, at least in part, to explain the observed reduction of the side effects of anti-cancer treatments if the fasting mimic diet is simultaneously followed. On the contrary, cancer cells have a reverse effect (differential stress response) (114) with inhibition of stress response.

**Figure 5:**
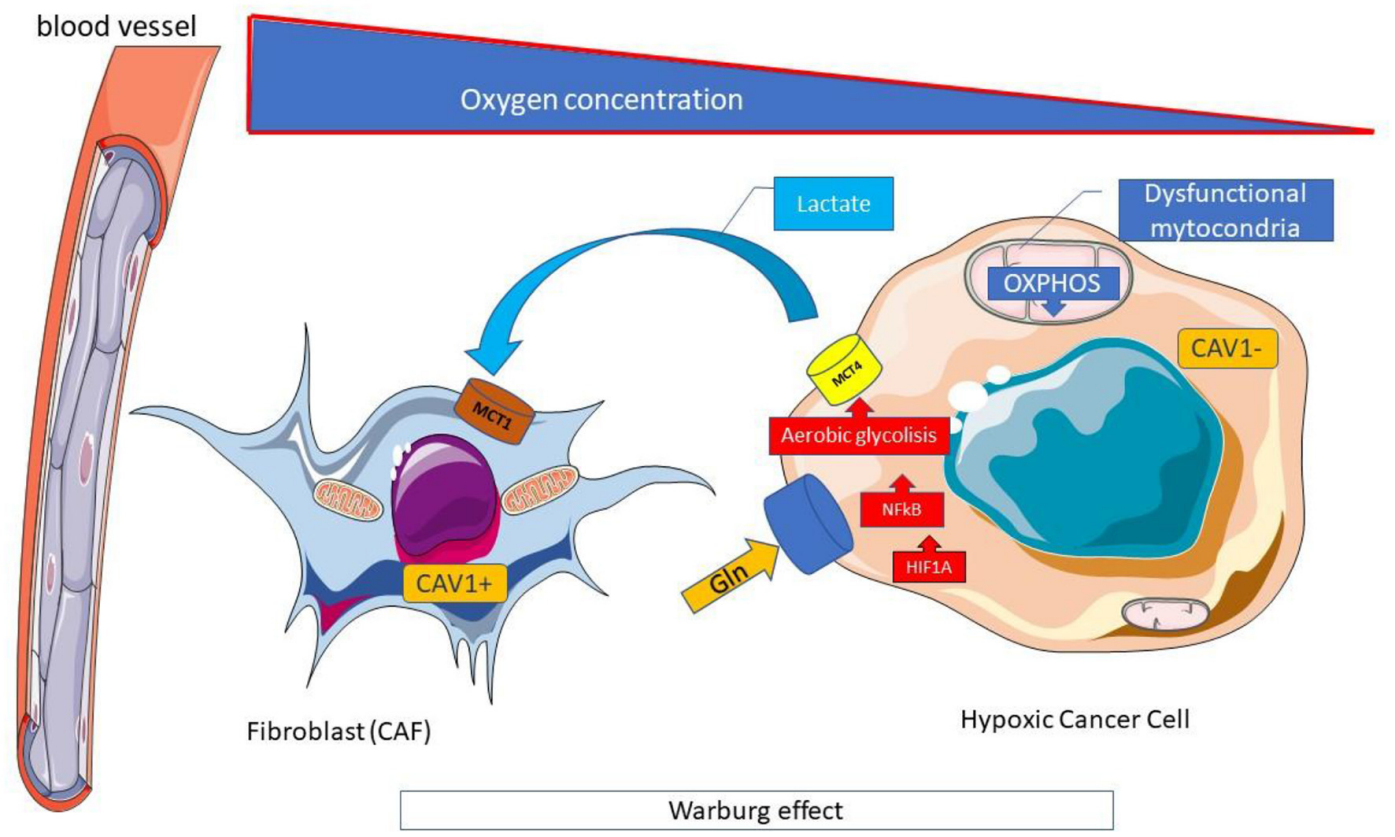
Warburg effect. The Warburg effect is a form of modified cell metabolism observed in many neoplastic cells where, unlike normal differentiated cells, which rely primarily on mitochondrial oxidative phosphorylation to generate the energy needed for cellular processes, relies on aerobic glycolysis. Aerobic glycolysis is an apparently inefficient way of generating adenosine 5'-triphosphate (ATP), compared to oxidative phosphorylation which allows the production of ATP by the oxidative disruption of pyruvate in mitochondria. However, the rate of glucose metabolism through aerobic glycolysis is higher, so that lactate production from glucose takes place 10–100 times faster than complete glucose oxidation in mitochondria. The metabolic difference observed by Warburg adapts cancer cells to hypoxic (oxygen deficient) conditions within solid tumors and derives largely from the same mutations of oncogenes and tumor suppressor genes that cause the other abnormal characteristics of cancer cells. Hypoxic conditions could induce HIF1A, a major regulator of glucose metabolism, and activate the expression of key enzymes for glycolysis. The high use of glucose in aerobic glycolysis, in addition to the high production of ATP, and the increase in the pathway of pentose phosphates, both essential for the anabolic processes necessary to support cell proliferation, is associated with the production of high levels of lactate and the acidification of the tumor microenvironment which plays a favorable role to the growth of neoplastic. CAV, Caveolin; Gln, Glutamine; MCT, Monocarboxylated transporter; OXPHOS, Oxidative phosphorylation.

The reduction in the availability of glucose in neoplastic cells determined by FMD causes a switch from aerobic glycolysis (Warburg effect) (Figure 5) toward oxidative phosphorylation and beta-oxidation of fatty acids, a necessary condition to allow cell growth in a nutrient-poor environment (115).

**Figure 6:**
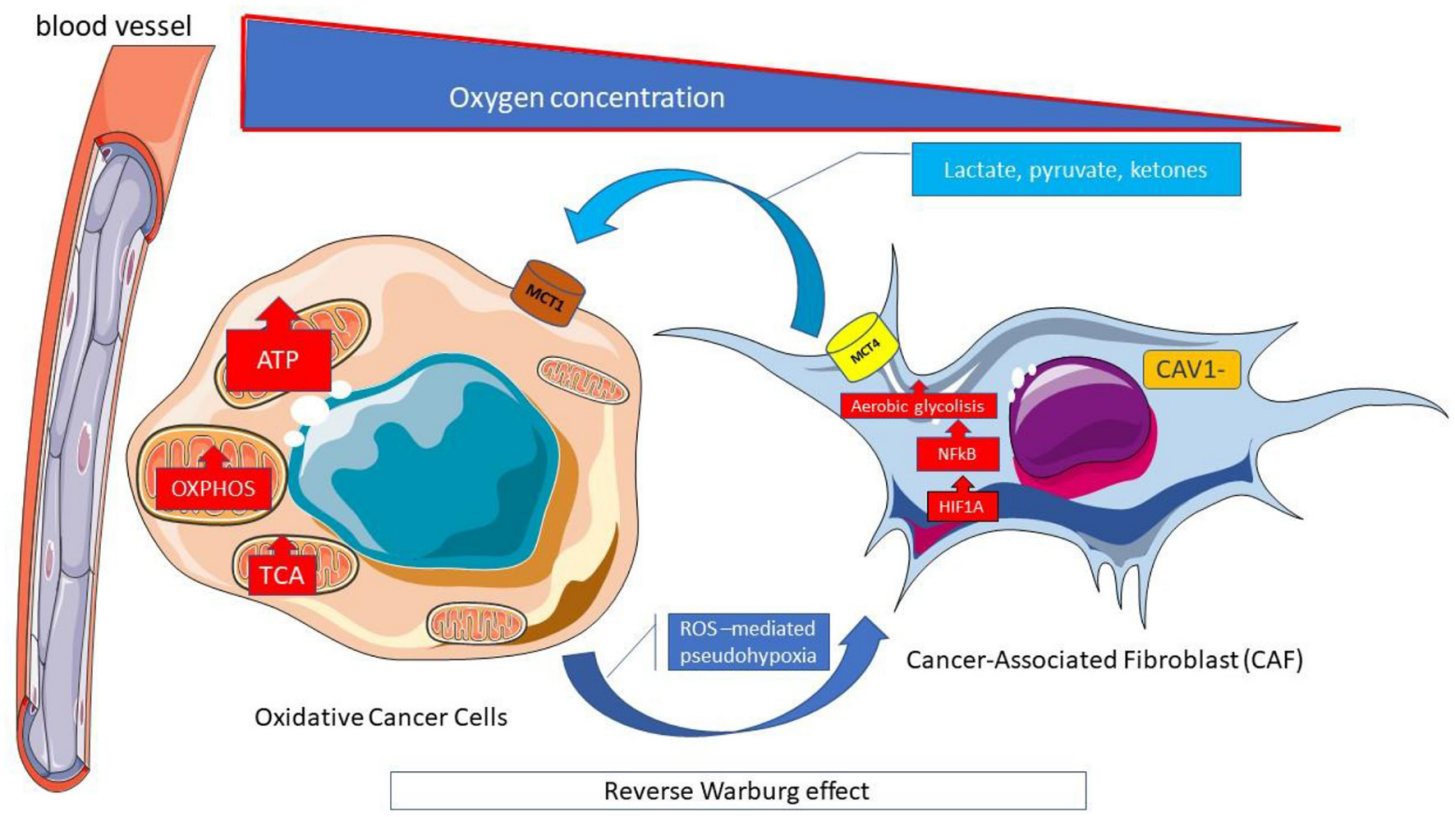
Reverse Warburg effect. Cells within tumors interact metabolically with the transfer of catabolites from supporting stromal cells to adjacent tumor cells. The reverse Warburg effect describes when the aerobic glycolysis of stromal fibroblasts associated with cancer metabolically supports adjacent cancer cells. The stromal-cancer metabolic coupling, allows cancer cells to generate ATP from substrates (lactate, pyruvate, ketonic bodies) provided by stromal cells, increase proliferation and reduce apoptosis. The Monocarboxylated transporter 4 MCT4 transporter is involved in the release of monocarboxylates from the tumor-associated fibroblast. It is regulated by catabolic transcription factors such as hypoxia inducible factor 1 alpha (HIF1A) and kappa-light-chain-enhancer nuclear factor of activated B-cells (NF-κB), and is highly expressed in cancer-associated fibroblasts. In contrast, MCT1 allows the absorption of these catabolites by neoplastic cells where it is highly expressed. ATP, Adenosine triphosphate; CAV, Caveolin; HIF1A; Hypoxia-inducible factor 1-alpha; MCT, Monocarboxylated transporter; NF-κB, Nuclear factor kappa B; OXPHOS, Oxidative phosphorylation; ROS, Reactive oxygen species; TCA, Tricarboxylic acid.

The increase in beta-oxidation in the mitochondria, in turn, causes an increase in ROS production and, at the same time a decrease in the cell's antioxidant defenses (glutathione) occurs; the two processes amplify oxidative stress and promote the activity of chemotherapy (115). There are, however, many doubts about this, as the Warburg effect in many cell lines can be thwarted by what is referred to as the “reverse Warburg effect” (Figure 6): a new model of cancer metabolism, in which cancer cells breathe by feeding on lactic acid produced by neighboring fibroblasts (116).

Indeed, in normal tissues, glucose is converted to pyruvate and transported to mitochondria for oxidative phosphorylation (OXPHOS). In Many types of tumors, some cancer cell (in particular cancer stem cells) express high levels of Caveolin 1 (Cav1) which regulates genes encoding glycolytic enzymes, glucose transporters and stimulate glycolysis, irrespective of the presence of oxygen without ATP generation by mitochondria (aerobic glycolysis or Warburg effect), in a crosstalk with Hypoxia-inducible factor 1-alpha (HIF-1α) (117, 118).

Other cancer cells (observed in breast, ovarian, prostate, liver, colon, pancreatic, and head and neck squamous cell cancer) can reprogram cancer-associated fibroblasts (CAF) that express monocarboxylate transporter 4 (MCT4) to undergo aerobic glycolysis and secrete energy-rich nutrients (lactate, pyruvate, beta-hydroxybutyrate, acetate). The CAF is obliged to feed cancerous cells that express MCT1 and have a high mitochondrial oxidative metabolism (reverse Warburg effect). These cancer cells overexpress monocarboxylate transporter1 (MCT1) (Figure 6). High stromal levels of MCT1 expression in cancer cells and high MCT4 expression in the stroma are specifically associated with poor overall survival (119).

MCT 4 expression in CAF starts from the production of reactive oxygen species (ROS) by cancer cells which freely spread in the microenvironment and enter into the adiacent CAF, causing oxidative stress (116). Oxidative stress leads to the activation of HIF-1α and NFκB (ROS-mediated pesudo-hypoxia) (Figure 5). HIF-1α triggers angiogenesis and aerobic glycolysis and moreover causes the loss of stromal Caveolin 1 (Cav-1) that amplifies oxidative stress through a “positive feedforward control” (Figure 6).

**Figure 7:**
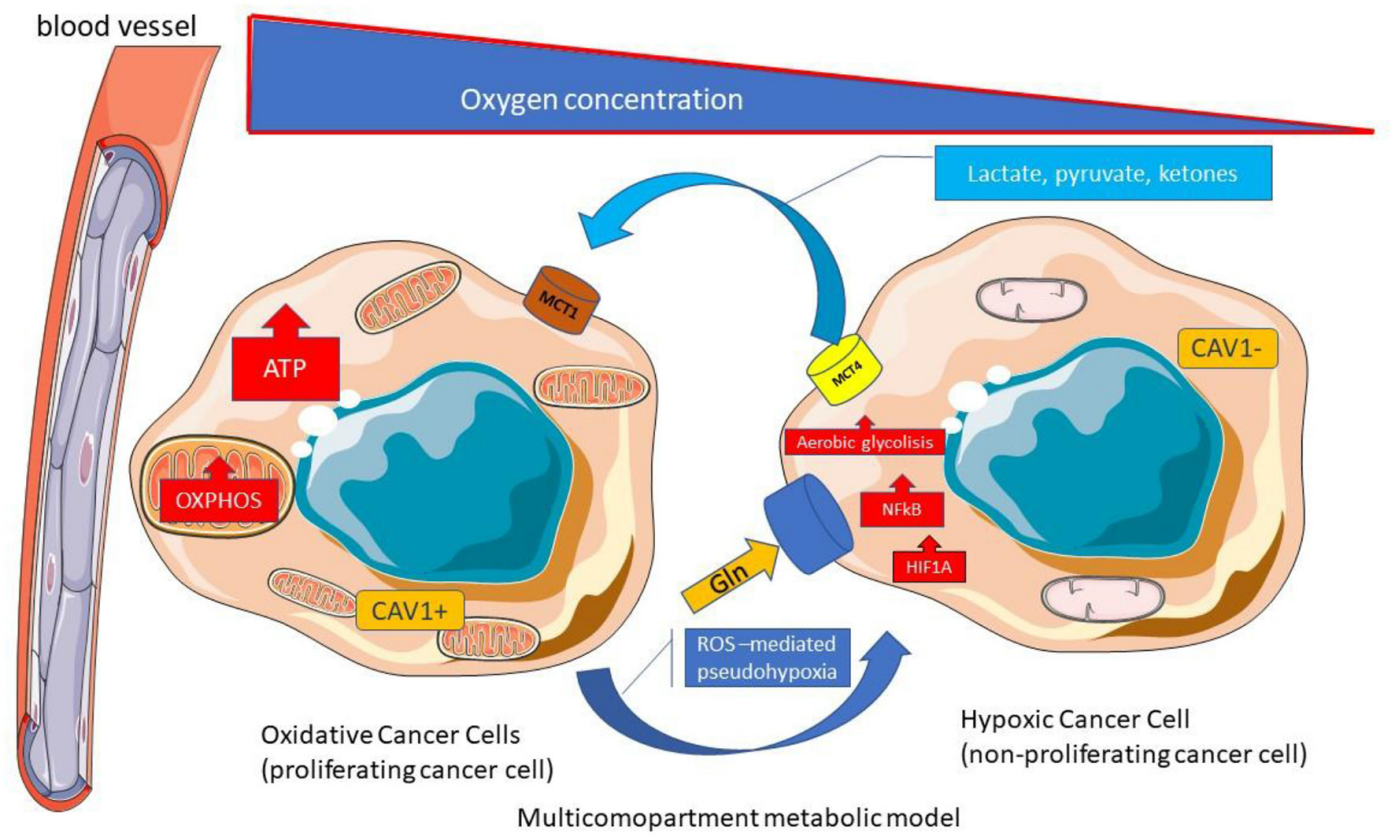
Multicompartment metabolic model. Multicompartimental metabolism model is similar to the reverse Warburg effect. In the multicompartimental model, the cell compartment of cancer cells is divided into a very proliferative compartment (consisting of oxidative cancerous cells with MCT1 expression) and a less proliferative compartment (consisting of hypoxic cancer cells). ATP, Adenosine triphosphate; CAV, Caveolin; Gln, Glutamine; HIF1A, Hypoxia-inducible factor 1-alpha; MCT, Monocarboxylated transporter; NF-κB, Nuclear factor kappa B; OXPHOS, Oxidative phosphorylation; TCA Tricarboxylic acid; ROS; Reactive Oxygen species.

The microenvinronmental cancer metabolism could be more complex (120). As a matter of fact a Multicompartment Metabolism Model is described similarly to the Reverse Warburg Effect. In this multicompartment model the cancer cell compartment is divided into a highly proliferative population (oxidative cancer cells with MCT1 expression) and a relatively less proliferative population (Hypoxic cancer cells) (Figure 7).

The CAF and hypoxic cancer cells with low proliferation rates guarantee nutrients for proliferative cancer cells in the Multicompartment Metabolism Model (119). The authors suggest that multiple types of metabolism models could be found in the different cancer phenotypes and even into different areas within a single tumor (119).

Furthermore, the hypothesis of using calorie and protein restriction cannot always be used in all types of cancer. Leucine and glucose intake could provoke in some cancer type a less activation of many anabolic pathways and cell proliferation, by inhibiting apoptosis and the activation of mTOR-c1. In this regard the reduction of leucine and glucose do not stimulate growth factor receptors (VEGF, IGF-1, EGF, etc.) and insulin, without activating phosphatidylinositol-3-kinase (PI3K) and AKT.

In fact, in some tumors the PI3K/AKT/mTOR pathway can be hyperactivated regardless of endocrine signals or the availability of nutrients. Cancer cell lines that present the PI3K mutation are in fact unresponsive to the effect of food energy restriction (120).

Studying a type of diet such as DMD/FMD, which provides only a few days a month of calorie restriction, could be a new complementary approach to standard drug therapies in the treatment of cancer; a cyclic fast of this type could be well-accepted by patients and if the data were to reflect the results on experimental animals, it could increase the tolerability and efficacy of chemotherapy agents and reduce side effects. It seems that this can be a feasible, well-tolerated and relatively safe dietary treatment, but it is essential to wait for convincing results from controlled clinical studies and large cases, in order to prevent the risk of aggravation of malnutrition which is instead of high frequency in many patients oncology.

Indeed, one of the main problems related to the applicability of these types of dietary regimens in humans, and in particular in cancer patients, is the high risk of exacerbating nutritional deficiencies, malnutrition, weight loss and sarcopenia related to the cancer itself and to anticancer treatments, at least in predisposed or fragile patients. Therefore, a comprehensive nutritional assessment must be performed before including cancer patients in a clinical trial with a fasting regimen and close clinical monitoring for the entire clinical trial is equally essential.

Recently, under an accurate nutritional control periodic fasting or FMD increases the anti-cancer activity of tamoxifen and fulvestrant, delays resistance to these agents and, in combination with fulvestrant and palbociclib, causes tumor regression and reverses acquired resistance to these two drugs. A pivotal cause for the enhancement of ET anti-tumor activity by fasting or FMD appears to be the reduction in blood insulin, IGF1 and leptin, with the consequent inhibition of the PI3K–AKT–mTOR pathway, at least in part through the upregulation of EGR1 and PTEN (121). These results have been obtained thank's to a multimodality nutritional treatment: alternating FMD cycle with oral nutritional supplement (121).

Malnutrition related to the disease is one of the main causes of death and morbidity in patients with cancer, in these cases cachexia leads to weight loss and muscle wasting. The incidence and prevalence of malnutrition in patients are between 40 and 80%, so it is a very serious and by no means negligible problem (122).

It is now widely known that nutritional parameters, and in particular body composition such as phase angle (PhA), a parameter closely related to the severity of cancer malnutrition, represents an important predictor of reduced survival in cancer patients undergoing anticancer treatments (123, 124).

## Author Contributions

SS was responsible for the conceptualization and design of the study and writing the manuscript. MM was responsible for the conceptualization and design and review the manuscript. All authors contributed to the article and approved the submitted version.

## Conflict of Interest

The authors declare that the research was conducted in the absence of any commercial or financial relationships that could be construed as a potential conflict of interest. The reviewer CL declared a shared affiliation with one of the authors MM to the handling editor at time of review.
